# The Neoepitopes on Methylglyoxal- (MG-) Glycated Fibrinogen Generate Autoimmune Response: Its Role in Diabetes, Atherosclerosis, and Diabetic Atherosclerosis Subjects

**DOI:** 10.1155/2021/6621568

**Published:** 2021-12-21

**Authors:** Shahnawaz Rehman, Jiantao Song, Mohammad Faisal, Abdulrahman A. Alatar, Firoz Akhter, Saheem Ahmad, Bo Hu

**Affiliations:** ^1^Department of Biochemistry, Sir Syed Faculty of Science, Mohammad Ali Jauhar University, Rampur, U.P., India; ^2^Department of Emergency, Shandong Provincial Hospital Affiliated to Shandong First Medical University, Jinan, Shandong 250021, China; ^3^Department of Botany & Microbiology, College of Science, King Saud University, P.O. Box 2455, Riyadh 11451, Saudi Arabia; ^4^Department of Biomedical Engineering, Stony Brook University, New York, USA; ^5^Department of Medical Laboratory Sciences, College of Applied Medical Sciences, University of Hail, 2440, Saudi Arabia

## Abstract

**Objectives:**

In diabetes mellitus, hyperglycemia-mediated nonenzymatic glycosylation of fibrinogen protein plays a crucial role in the pathogenesis of micro- and macrovascular complications especially atherosclerosis via the generation of advanced glycation end products (AGEs). Methylglyoxal (MG) induces glycation of fibrinogen, resulting in structural alterations that lead to autoimmune response via the generation of neoepitopes on protein molecules. The present study was designed to probe the prevalence of autoantibodies against MG-glycated fibrinogen (MG-Fib) in type 2 diabetes mellitus (T2DM), atherosclerosis (ATH), and diabetic atherosclerosis (T2DM-ATH) patients. *Design and Methods*. The binding affinity of autoantibodies in patients' sera (T2DM, *n* = 100; ATH, *n* = 100; and T2DM-ATH, *n* = 100) and isolated immunoglobulin G (IgG) against native fibrinogen (N-Fib) and MG-Fib to healthy subjects (HS, *n* = 50) was accessed by direct binding ELISA. The results of direct binding were further validated by competitive/inhibition ELISA. Moreover, AGE detection, ketoamines, protein carbonyls, hydroxymethylfurfural (HMF), thiobarbituric acid reactive substances (TBARS), and carboxymethyllysine (CML) concentrations in patients' sera were also determined. Furthermore, free lysine and free arginine residues were also estimated.

**Results:**

The high binding affinity was observed in 54% of T2DM, 33% of ATH, and 65% of T2DM-ATH patients' samples with respect to healthy subjects against MG-Fib antigen in comparison to N-Fib (*p* < 0.05 to *p* < 0.0001). HS sera showed nonsignificant binding (*p* > 0.05) with N-Fib and MG-Fib. Other biochemical parameters were also found to be significant (*p* < 0.05) in the patient groups with respect to the HS group.

**Conclusions:**

These findings in the future might pave a way to authenticate fibrinogen as a biomarker for the early detection of diabetes-associated micro- and macrovascular complications.

## 1. Introduction

In diabetes mellitus, hyperglycemia-mediated nonenzymatic glycosylation or glycation of biological macromolecules (proteins, lipids, and DNA) plays a critical role in the pathogenesis of secondary complications especially micro- and macrovascular complications [1, 2]. During glycation, free carbonyl groups (>C=O) of reducing sugars bind with free amino (-NH_2_) groups at the N-terminal of proteins, *ε*-amino group of lysine, guanidino group of arginine, and imidazole group of histidine to form reversible aldimines or Schiff's bases [3, 4]. Schiff's bases via keto-enol tautomerism and acid-base catalysis convert to irreversible ketoamines or Amadori products, and the latter undergo rearrangements, dehydrations, and cyclizations to form heterogeneous molecular adducts, known as advanced glycation end products (AGEs) [5, 6].

Glycoxidation of glycation-adducts and autoxidation of glucose lead to the generation of reactive oxygen species (ROS) such as superoxide radicals (O_2_^•^) via transition metal-catalyzed reaction and hydroxyl radicals (^•^OH) via Fenton's reaction, thereby inducing oxidative stress [7, 8]. Besides ROS generation, both autoxidation and glycoxidation give rise to aldehyde and/or ketone groups possessing highly electronegative dicarbonyls, called reactive carbonyl species (RCS), i.e., glyoxal (GO), methylglyoxal (MG), and 3-deoxyglucosone (3-DG) [9, 10]. Moreover, ROS-mediated oxidative stress induces lipid peroxidation and oxidation of amino acids, which result in the generation of RCS, especially MG. Furthermore, channelization of excess glucose in glycolysis and the polyol pathway leads to the generation of MG [11, 12]. In human plasma, the physiological concentration of MG is 50-150 nM [13, 14]. When this concentration goes beyond the normal and remains unchecked, the cellular system suffers carbonyl stress [15]. Furthermore, MG similarly glycates proteins as that of reducing sugars, but this time more violently, and induces a glycation-oxidation vicious cycle, thus accelerating glycative, oxidative, and carbonyl stress, the necessary outcome of which is AGE formation [16–18].

Fibrinogen (340 kDa; half-life ~4 days; concentration 2-4 mg mL^−1^) is an independent marker for vascular complications especially in atherosclerosis [19, 20]. Elevated fibrinogen levels and glycative insult suffered by fibrinogen are among the factors that are responsible for atherosclerosis-mediated CVD risk in diabetic patients [21, 22]. The glycemic control influences the survival of fibrinogen in circulation that reduces during hyperglycemia and normalizes during euglycemia [23]. This correlation results due to the extent of fibrinogen glycation, as the glycated fibrinogen, clear more rapidly than nonglycated fibrinogen [24]. It has been reported that glycated fibrinogen usually resides extravascularly whereas the nonglycated one is distributed intravascularly. The extravascular accumulation (in the endothelial cells) of fibrinogen-AGEs (Fib-AGEs) results in their binding to receptors, called receptor for AGEs (RAGE), thereby establishing an AGE-RAGE axis. This axis triggers a signaling cascade, which in turn promotes atherosclerosis via the activation of ERK, JNK, PI3K, p38, NF-*κ*B, and cAMP pathways. Thus, the increased uptake of glycated fibrinogen into vascular walls might contribute to the progression of atherosclerosis in diabetic patients [25]. Glycation causes structural modifications and functional alterations in fibrinogen molecule that ultimately results in the formation of a lysis-resistant dense and less porous fibrin network [26].

The lysine residues of fibrinogen play a central role in the formation and degradation (fibrinolysis) of the fibrin network. During blood coagulation, fibrinopeptides A and B cleave from the fibrinogen molecule by the action of thrombin to form fibrin monomers. These monomers polymerize to form protofibrils and get interwoven to give rise to a fibrin network or clot. The tensile strength of the clot and its resistance to lysis are imparted by peptide bonds between glutamine and lysine residues. These peptide bonds between adjacent fibrin molecules are formed by the action of activation factor XIII [25]. The lysis of the fibrin network (clot) is carried either by activation factor XIII-induced cross-linkage at the lysine residue or by direct binding of plasminogen, tissue plasminogen activator (tPA), and plasminogen activator inhibitor-1 (PAI-1) to lysine residues in fibrin molecules [27, 28]. Thus, the glycation at lysine residues affects factor XIII cross-linkage, fibrin clot structure, and regulation of fibrinolysis. Glycated fibrinogen possesses higher clotting activity than nonglycated fibrinogen [29]. It has been reported that resistance to fibrinolysis increases with an increase in fibrinogen glycation [30]. The plasminolysis system that comprises plasminogen, tPA, and PIA-1 requires to bind at free lysine residues for lysis. However, lysine residues are engaged due to glycation and are not available for the process of fibrinolysis [31, 32].

Moreover, AGE accumulation results in the monocyte-macrophage-mediated production of cytokines (IL-1 and IL-6), which in turn activates hepatocytes for the overproduction of fibrinogen [33, 34]. Besides the expression of adhesion molecules on endothelial cells, AGEs also stimulate monocyte migration and cytokine secretion from macrophages [35]. Furthermore, AGEs increase the levels of endothelin-1 and decrease the levels of nitric oxide, thereby resulting in overall vasoconstriction [36]. Additionally, AGEs facilitate clot formation by suppressing fibrinolysis via the activation of plasminogen activator inhibitor-1 (PAI-1) [37].

Glycation of fibrinogen with MG results in structural modifications and exposure of neoepitopes that elicit immune responses in experimental animals [38]. Previous studies by our research group have revealed the presence of autoantibodies against d-ribose-glycated LDL, d-ribose-glycated hemoglobin (Hb), and MG-glycated LDL in type 2 diabetes mellitus (T2DM) and its associated secondary complications [39–44]. Therefore, we hypothesized that MG-mediated glycation of fibrinogen leads to the generation of autoantibodies (anti-MG-Fib-IgG) in a hyperglycemic state. The present study was designed to probe the prevalence of autoantibodies against MG-glycated fibrinogen (MG-Fib) in patients suffering from T2DM, atherosclerosis (ATH), and diabetic atherosclerosis (T2DM-ATH).

## 2. Materials and Methods

### 2.1. Materials

Fibrinogen, methylglyoxal, oxalic acid, thiobarbituric acid (TBA), trichloroacetic acid (TCA), sodium chloride (NaCl), sodium hydroxide (NaOH), phosphotungstic acid, bovine serum albumin (BSA), ammonium persulphate (APS), bis-acrylamide, and tetramethylethylenediamine (TEMED) were purchased from Hi-Media. Sodium dodecyl sulfate (SDS) and 2,4,-trinitrobenzene-1-sulphonic acid (TNBS) were obtained from G Biosciences, whereas ethyl acetate and dinitrophenylhydrazine (DNPH) were purchased from Rankem. Guanidium hydrochloride was obtained from S. D. Fine-Chem Limited, and hydrochloric acid (HCl) was purchased from Fisher Scientific. A protein A agarose column was purchased from Sigma, and polystyrene plates were obtained from Nunc (Denmark). Analytical grade chemicals were used in this study. All the solutions were filtered under aseptic conditions using a sterilized Puradisc™ 0.2 mM syringe filter (Whatman, GE Healthcare UK Limited, UK).

#### 2.1.1. Ethical Statement

The clinical study has been approved by the “Institutional Ethics Committee (IEC) of Integral Institute of Medical Sciences and Research” (IIMS&R), Lucknow, U.P., India, with approval number IEC/IIMS&R/2019/51.

#### 2.1.2. Population Study and Collection of Sera

The study was performed on 350 serum samples from patients and healthy individuals. The study subjects were divided into four groups, i.e., (1) healthy subjects (HS, *n* = 50), (2) type 2 diabetes mellitus (T2DM, *n* = 100), (3) atherosclerosis (ATH, *n* = 100), and (4) diabetic atherosclerosis (T2DM-ATH, *n* = 100). Prior consent as per the guidelines of the Helsinki declaration was taken ahead of blood withdrawal from healthy subjects and patients. The blood samples were centrifuged at 4°C for 20 minutes at 2000 rpm. Isolated serum samples were incubated for 30 min at 56°C to inactivate complement proteins. Finally, 0.01% sodium azide was added to the sera, and the latter were stored at -20°C. Serum samples from the HS group were used as a control.

#### 2.1.3. Inclusion/Exclusion Criteria

*(1) Inclusion Criteria*.

- Patients with T2DM, ATH, and T2DM-ATH
- Age group between 40 and 75 years with disease duration 5-15 years for all patients

*(1) Exclusion Criteria*.

- Patients suffering from any chronic disease other than T2DM, ATH, and T2DM-ATH
- Patients suffering from any acute or chronic infection

#### 2.1.4. Biochemical Investigations

The clinical data of biochemical parameters such as BMI, WHR, blood pressure (systolic/diastolic), fasting blood glucose, HbA1c, total cholesterol (TC), triglycerides (TG), low-density lipoprotein (LDL), and high-density lipoprotein (HDL) were collected from the clinical laboratory of IIMS&R, Lucknow, India.

### 2.2. Methods

#### 2.2.1. Preparation of Antigen: Methylglyoxal-Glycated Fibrinogen

Human fibrinogen (150 *μ*g mL^−1^) was treated with 10 mM of methylglyoxal (MG) in 100 mM sodium phosphate buffer saline (PBS) (7.4 pH) under sterile conditions. Sodium azide (0.05%) was added to reaction solutions to inhibit fungal and microbial growth. Both the native fibrinogen (N-Fib) and MG-glycated fibrinogen (MG-Fib) were incubated at 37°C for 7 days. Extensive dialysis was performed to get rid of unbound constituents. The MG-induced structural modifications in MG-Fib against N-Fib were characterized by different biochemical and biophysical techniques as previously published by us [38].

#### 2.2.2. Direct Binding ELISA of Patients' Sera

Direct binding ELISA was accomplished on a 96-well polystyrene plate to validate titer of autoantibodies that might have risen against N-Fib and MG-Fib in T2DM, ATH, and T2DM-ATH patients [45]. The wells were coated with 10 *μ*g of N-Fib and MG-Fib in 100 *μ*L of 0.5 mol L^−1^ carbonate/bicarbonate buffer having a pH of 9.6. The samples were coated in triplicate, and half part of the ELISA plate was used as the control. The plates were incubated at 37°C (2 h) and were left at 4°C overnight. After overnight incubation, washing was done thrice with TBS-T (20 mM Tris, 2.68 mM KCL, and 150 mM NaCl (pH 7.4) having 0.05% Tween-20). The unoccupied sites of wells were blocked with 2.5% bovine serum albumin (BSA), and the plates were again incubated at 37°C (4 h). After incubation, plates were again washed thrice with TBS-T. Serially diluted sera (1 : 100) from control and patients were appended to each well (100 *μ*L per well), and the plates were incubated at 37°C (2 h). After incubation, the wells were again washed thrice with TBS-T and were coated with anti-human IgG-alkaline phosphatase (1 : 2000) and left for two hours at 37°C. After incubation, washing was done with TBS-T and the wells were coated with *p*-nitrophenylphosphate (pNPP) substrate. The reading was taken at 410 nm on an ELISA plate reader, and the results were calculated as a mean of *A*_test_ − *A*_control_.

#### 2.2.3. IgG Isolation from HS and T2DM, ATH, and T2DM-ATH Patients' Sera

The protein A agarose affinity column was used to isolate immunoglobulin G (IgG) from HS and T2DM, ATH, and T2DM-ATH patients' sera that showed high autoantibody titer in the direct binding ELISA. Equal volumes (0.5 mL) of serum and phosphate buffer saline (0.1 M; pH 7.4) were mixed and passed through the affinity column 3-5 times. The bound IgG was eluted by 0.058% acetic acid in 0.85% sodium chloride solution. The 2 mL eluted solution containing IgG was collected in 1 mL of 1 M Tris-HCl (pH 8.5) buffer. The concentration of isolated IgG was determined by using the molar extinction coefficient of 2.1 × 10^5^ M^−1^ cm^−1^ at 280 nm wavelength [46].

#### 2.2.4. Direct Binding ELISA of Isolated IgG

Direct binding ELISA was performed to elucidate the binding affinity of IgG that was purified from T2DM, ATH, and T2DM-ATH patients' sera. Polystyrene plates were coated with the N-Fib and MG-Fib (10 *μ*g/100 *μ*L). The experimental procedure was the same as described for ELISA of the patients' sera (Section 2.2.2) except that purified IgG was used in place of sera [47].

#### 2.2.5. Competitive ELISA

Competitive/inhibition ELISA was performed to ensure antigen-binding specificity of autoantibodies that were generated against N-Fib and MG-Fib in patients' sera. Immune complexes were prepared by mixing antigens (N-Fib and MG-Fib) in 0.1, 0.5, 1, 5, 10, 20, 50, and 100 *μ*g mL^−1^ concentrations with a constant amount of purified autoantibodies and were incubated at 37°C for two hours and then left at 4°C overnight. Native immune complexes (N-Fib+N-Fib-IgG) and glycated immune complexes (MG-Fib+MG-Fib-IgG) were used as inhibitors. All steps were the same as discussed earlier in the direct binding ELISA except that immune complexes were used as primary antibodies to be coated in place of sera or IgG [48]. Percent inhibition was calculated as

(1) $$\begin{array}{c} \text{Percent inhibition}\left(\%\right)=\frac{1‐\text{inhibited}}{\text{Uninhibited}}\times 100. \end{array}$$

#### 2.2.6. Nitroblue Tetrazolium (NBT) Reduction Assay

The nitroblue tetrazolium (NBT) reduction assay was performed to detect ketoamines in the sera of HS and T2DM, ATH, and T2DM-ATH patients. The serum samples (20 *μ*L) were mixed with 180 *μ*L of Na_2_CO_3_/NaHCO_3_ buffer (100 mM, pH 10.8) containing 0.25 mM NBT. The mixtures were incubated at 37°C until the color changed from yellow to purple. The absorbance was recorded at 525 nm, and the molar extinction coefficient of 12,640 M^−1^ cm^−1^ was used to calculate ketoamine concentration (nM mL^−1^) [49, 50].

#### 2.2.7. Fluorescence Spectroscopy

Fluorescence spectroscopy was performed to detect fluorescent AGEs in the sera of HS and T2DM, ATH, and T2DM-ATH patients. The 0.1 mL of serum was mixed with 0.4 mL of 100 mM PBS of pH 7.4. The sample was excited at 370 nm, and the emission was recorded between 400 and 500 nm on an Agilent Cary Eclipse fluorescence spectrophotometer at 25 ± 0.2°C in a 1 cm path length quartz cuvette. The slit width was set at 5 nm [51, 52]. The percent increase in fluorescence intensity (F.I.) was calculated by the following equation:

(2) $$\begin{array}{c} \%\text{Increase in F}.\text{I}.=\frac{\text{F}.\text{I}.\text{of patien}{\text{t}}^{\prime }\text{s serum}–\text{F}.\text{I}.\text{of HS serum}}{\text{F}.\text{I}.\text{of patien}{\text{t}}^{\prime }\text{s serum}}\times 100. \end{array}$$

#### 2.2.8. Protein Carbonyl Estimation

Protein carbonyl estimation in the sera of HS and T2DM, ATH, and T2DM-ATH patients was performed by using 2,4-dinitrophenylhydrazine (DNPH). The 0.1 mL of serum was mixed with 0.5 mL of 10 mM DNPH in 2.5 N HCL and was incubated at room temperature for 1 hour. After incubation, 0.5 mL of TCA (20% *v*/*v*) was added and the mixture was centrifuged at 10,000 rpm for 10 minutes to precipitate DNP hydrazones. The precipitated pellets were extensively washed three times with 0.5 mL of ethanol-ethyl acetate solution (1 : 1 *v*/*v*) to relieve surplus DNPH. The pellets were then dissolved in 0.25 mL of 6 M guanidium hydrochloride solution. Protein carbonyl concentration (nM mg^−1^) was evaluated by using a molar extinction coefficient of 22,000 M^−1^ cm^−1^ at 370 nm wavelength [53, 54].

#### 2.2.9. Hydroxymethylfurfural (HMF) Estimation

Hydroxymethylfurfural (HMF) estimation in the sera of HS and T2DM, ATH, and T2DM-ATH patients was performed by using thiobarbituric acid (TBA). The 0.1 mL of serum was mixed with 0.1 mL of 1 M oxalic acid and was left for 1 hour in the water bath. After cooling at room temperature, trichloroacetic acid (40%) was mixed in the solutions. The precipitate was discarded, and the thiobarbituric acid (TBA) was added to the supernatant and was incubated at 37°C for half an hour. After the appearance of color, HMF concentration (nM mL^−1^) was evaluated by using the molar extinction coefficient of 4 × 104 M^−1^ cm^−1^ at 443 nm wavelength [55].

#### 2.2.10. Thiobarbituric Acid Reactive Substance (TBARS) Estimation

TBARS estimation in the sera of HS and T2DM, ATH, and T2DM-ATH patients was performed by adding H_2_SO_4_ and phosphotungstic acid. The 0.1 mL of serum was mixed with 0.4 mL of 0.083 N H_2_SO_4_ and 0.2 mL of 10% phosphotungstic acid. The solutions were left at room temperature for 5 minutes and then centrifuged at 3000 rpm for 10 minutes. The pellets were mixed with 2 mL of distilled water, and 0.5 mL of TBA reagent (a mixture containing 0.37% aqueous TBA, 15% aqueous TCA, and 0.25 N HCl) was added. The solutions were heated for 1 hour at 95°C, and centrifugation at 3000 rpm for 10 minutes was performed to collect the supernatant. The supernatant was left to cool at room temperature. TBARS concentration (*μ*M mL^−1^) was evaluated by using the molar extinction coefficient of 1.56 × 10^5^ M^−1^ cm^−1^ at 532 nm wavelength [56].

#### 2.2.11. Free Lysine Residue Estimation

Free lysine residue estimation in the sera of HS and T2DM, ATH, and T2DM-ATH patients was performed by using 2,4,6-trinitrobenzene sulphonic acid (TNBS). The 0.1 mL serum was mixed with 0.1 mL of NaHCO_3_ buffer (4% *w*/*v*, pH 8.5). The 0.1 mL of 0.1% TNBS was added to the solutions, and the latter were incubated at 40°C for 2 hours. After incubation, 0.45 mL of HCL was added and again the solutions were incubated at 110°C for 90 minutes. After cooling the solutions, centrifugation was done at 3000 rpm for 10 minutes. The supernatant was mixed with 1 mL ether to remove the *α*-TNP amino complex, and finally the solutions were kept on a water bath till the evaporation of excess ether. The absorbance was taken at 420 nm [57, 58].

#### 2.2.12. Free Arginine Residue Estimation

Free arginine residue estimation in the sera of HS and T2DM, ATH, and T2DM-ATH patients was performed by using 9,10-phenanthrenequinone. The 0.1 mL serum was mixed with 0.3 mL of 200 *μ*M of phenanthrenequinone (dissolved in ethanol). After mixing, 0.5 mL of 2 N NaOH was added and the solutions were left for 60 min at 30°C. After cooling at room temperature, 0.5 mL of 1.2 M HCl was added and the fluorescence was taken on the Agilent Cary Eclipse fluorescence spectrophotometer at 25 ± 0.2°C in a 1 cm path length quartz cuvette. The samples were excited at 312 nm, and the emission was recorded at 395 nm [59].

#### 2.2.13. Detection of Nonfluorogenic AGE: Carboxymethyllysine (CML)

Carboxymethyllysine (CML) is a nonfluorogenic AGE that was detected in the sera of HS and T2DM, ATH, and T2DM-ATH patients by using a sandwich ELISA kit from Bioassay Technology Laboratory (Korain Biotech Co. Ltd.). The serum was diluted to 1 : 100 ratios with PBS (100 mM; pH 7.4). The diluted samples were added to the wells that were already coated with monoclonal antibodies against CML. After incubating for 15 minutes, the anti-CML antibody was added. The excess anti-CML antibody was removed during the washing step. Later on, streptavidin-HRP was added to the wells that bound to the anti-CML antibodies. After incubating for 15 minutes, washing was done to remove unbound streptavidin-HRP. Finally, the substrate was added that imparted color in the proportion of CML present in the serum samples. After the appearance of color, the reaction was terminated by adding an acidic stop solution and the absorbance was read at 450 nm [60].

#### 2.2.14. Statistical Analysis

The data for the present study was presented as mean ± SD, and the statistical significance was determined by one-way ANOVA followed by the post hoc test, Tukey's multiple comparison test, and Dunnett *t*-test using the Statistical Package for the Social Sciences (SPSS) version 20.0. The coefficient of variation within groups (HS, T2DM, ATH, and T2DM-ATH) was analyzed by an unpaired *t*-test. The value of *p* < 0.05 was considered statistically significant. The scattered plots were plotted using GraphPad Prism version 5.0.1.

## 3. Results

### 3.1. Biophysical and Biochemical Characterization of Methylglyoxal-Glycated Fibrinogen

The *in vitro* methylglyoxal- (MG-) mediated glycation of fibrinogen protein results in secondary and tertiary level structural modifications as illustrated in our previous study [38]. The results of physicochemical characterization of native fibrinogen (N-Fib) and MG-glycated fibrinogen (MG-Fib) are summarized in Table 1.

### 3.2. Evaluation of Clinical Parameters in Patients and Healthy Subjects

The clinical parameters of HS and T2DM, ATH, and T2DM-ATH patients vary significantly in age and gender distribution at the time of testing. Gender difference does not play a significant role in the levels of MG-glycated fibrinogen levels in the patients. However, factors besides glycation such as smoking, alcohol consumption, disturbed daily routine, lifestyle, obesity, depression, hypertension, and stress are responsible for the gender-dependent development of cardiovascular diseases. Blood pressure (mmHg), both systolic (SST) and diastolic (DST), was moderately higher in T2DM patients (125 ± 12/78 ± 9) but was in the normal range, whereas in ATH (164 ± 22/106 ± 10) and T2DM-ATH (180 ± 9.5/110 ± 12) patients, the blood pressures were significantly higher with respect to HS (120 ± 15/65 ± 7). The body mass index (BMI) of HS (20.5 ± 1.5 kg m^−2^) and ATH patients (21.6 ± 2.0 kg m^−2^) was almost similar. However, in T2DM (26.55 ± 2.13 kg m^−2^) and T2DM-ATH (27.3 ± 2.3 kg m^−2^) patients, BMI was significantly higher with respect to HS. The HbA1c was found to be significantly higher in T2DM (6.6 ± 1.92%) and T2DM-ATH (7.0 ± 2.81%) patients than in ATH (4.4 ± 0.22%) patients and HS (4.5 ± 0.14%). The fasting glucose levels (mg dL^−1^) were significantly higher in T2DM (196 ± 8.2) and T2DM-ATH (222 ± 16) patients with respect to ATH (90 ± 6) and HS (88 ± 8). The lipid profiles of ATH and T2DM-ATH patients vary significantly with respect to T2DM and HS groups. The data of clinical parameters are presented in Table 2.

### 3.3. Direct Binding ELISA with Sera of T2DM, ATH, and T2DM-ATH Patients

**Figure 1:**
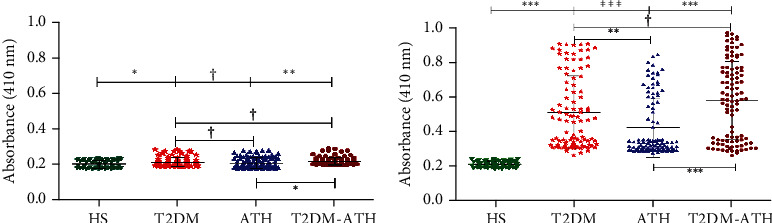
Direct binding ELISA of serum autoantibodies. (a) Direct binding ELISA of serum autoantibodies from diabetic (T2DM, *n* = 100), atherosclerosis (ATH, *n* = 100), and diabetic atherosclerosis (T2DM-ATH, *n* = 100) patients with respect to healthy subjects (HS, *n* = 50) against N-Fib (10 *μ*g mL^−1^). T2DM (^∗^*p* < 0.05) and T2DM-ATH (^∗∗^*p* < 0.001) groups show significant binding with respect to HS. The ATH group shows a nonsignificant (^†^*p* > 0.05) binding with respect to HS. Data are presented as mean ± SD of three independent experiments. (b) Direct binding ELISA of serum autoantibodies from diabetic (T2DM, *n* = 100), atherosclerosis (ATH, *n* = 100), and diabetic atherosclerosis (T2DM-ATH, *n* = 100) patients with respect to healthy subjects (HS, *n* = 50) against MG-Fib (10 *μ*g mL^−1^). All the three patient groups showed significant binding with respect to HS and among themselves (^∗^*p* < 0.05, ^∗∗^*p* < 0.001, and ^∗∗∗^*p* < 0.0001). Data are presented as mean ± SD of three independent experiments.

Direct binding ELISA was performed for the screening of HS and T2DM, ATH, and T2DM-ATH patients' sera against N-Fib and MG-Fib. The sera of the HS group were used as the control. The mean absorbance values at 410 nm of all the four groups were as follows: HS (0.204 ± 0.021 (0.175-0.234)), T2DM (0.215 ± 0.024 (0.192-0.288)), ATH (0.212 ± 0.029 (0.180-0.282)), and T2DM-ATH (0.220 ± 0.021 (0.200-0.295)). The autoantibodies in the sera of T2DM and T2DM-ATH groups showed significant binding against N-Fib with respect to the HS group, i.e., *p* < 0.05 and *p* < 0.001, respectively. However, between the HS group and the ATH group, the binding of autoantibodies against N-Fib was not significant (*p* > 0.05). Moreover, ATH and T2DM-ATH groups showed significance (*p* < 0.05) among them. The coefficient of variation in HS and T2DM, ATH, and T2DM-ATH groups was 10.63%, 11.50%, 13.78%, and 9.72%, respectively (Figure 1(a)).

The autoantibodies in the sera of T2DM (54%), ATH (33%), and T2DM-ATH (65%) exhibited higher binding affinity for MG-Fib. The mean absorbance values at 410 nm of all the four groups were as follows: HS (0.211 ± 0.018 (0.182-0.240)), T2DM (0.513 ± 0.209 (0.264-0.908)), ATH (0.424 ± 0.170 (0.280-0.846)), and T2DM-ATH (0.581 ± 0.223 (0.266-0.970)).

In the T2DM group, out of 54 samples that showed significant binding against MG-Fib, 17 samples were highly significant (*p* < 0.0001), 13 samples were very significant (*p* < 0.001), and 24 samples were significant (*p* < 0.05). In the ATH group, out of 33 samples, 7 samples were highly significant (*p* < 0.0001), 12 samples were very significant (*p* < 0.001), and 14 samples were significant (*p* < 0.05). Similarly, in the T2DM-ATH group, out of 65 samples, 21 samples were highly significant (*p* < 0.0001), 20 samples were very significant (*p* < 0.001), and 24 samples were significant (*p* < 0.05). With respect to HS, all the three patient groups exhibited high statistical significance (*p* < 0.0001). However, between T2DM and T2DM-ATH, the significance level was *p* > 0.05. The coefficient of variation in HS, T2DM, ATH, and T2DM-ATH groups was 8.81%, 40.70%, 40.09%, and 38.37%, respectively (Figure 1(b)). The statistical data are summarized in Table 3.

Note that after the screening, all the biophysical and biochemical experimentations were performed on 54 T2DM, 33 ATH, and 65 T2DM-ATH serum samples.

### 3.4. Direct Binding ELISA with IgG of T2DM, ATH, and T2DM-ATH Patients

**Figure 2:**
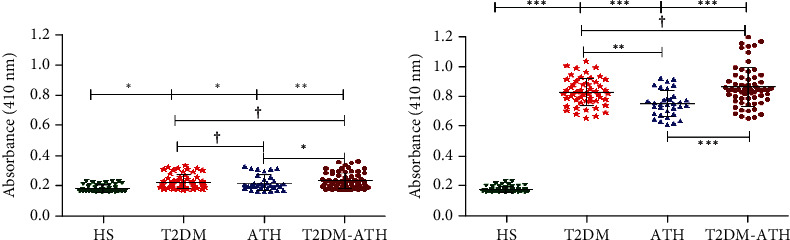
Direct binding ELISA of isolated IgG. (a) Direct binding ELISA of isolated IgG from diabetic (T2DM, *n* = 54), atherosclerosis (ATH, *n* = 33), and diabetic atherosclerosis (T2DM-ATH, *n* = 65) patients with respect to healthy subjects (HS, *n* = 50) against N-Fib (10 *μ*g mL^−1^). All the three patient groups showed significant binding (^∗^*p* < 0.05 and ^∗∗^*p* < 0.001) with respect to HS. Data are presented as mean ± SD of three independent experiments. (b) Direct binding ELISA of isolated IgG of diabetic (T2DM, *n* = 54), atherosclerosis (ATH, *n* = 33), and diabetic atherosclerosis (T2DM-ATH, *n* = 65) patients with respect to healthy subjects (HS, *n* = 50) against MG-Fib (10 *μ*g mL^−1^). All the three patient groups showed a significant binding affinity with respect to HS and among themselves (^∗^*p* < 0.05, ^∗∗^*p* < 0.001, and ^∗∗∗^*p* < 0.0001), except between T2DM and T2DM-ATH (^†^*p* > 0.05). Data are presented as mean ± SD of three independent experiments.

Direct binding ELISA with purified IgG from patients' sera was performed to further validate the binding affinity of autoantibodies against N-Fib and MG-Fib. The IgG was purified from the serum samples that showed higher binding during screening (Section 3.3). Direct binding of purified IgG with N-Fib showed statistical significance in T2DM (*p* < 0.05), ATH (*p* < 0.05), and T2DM-ATH (*p* < 0.001) with respect to the HS group (Figure 2(a)). However, between ATH and T2DM-ATH, the significance level was *p* < 0.05. The mean absorbance values at 410 nm of all the four groups against N-Fib were as follows: HS (0.186 ± 0.022 (0.163-0.235)), T2DM (0.231 ± 0.046 (0.178-0.338)), ATH (0.225 ± 0.050 (0.167-0.330)), and T2DM-ATH (0.238 ± 0.050 (0.180-0.370)). The coefficient of variation in HS, T2DM, ATH, and T2DM-ATH groups was 12.16%, 20.19%, 22.31%, and 21.85%, respectively.

In the direct binding of IgG with MG-Fib, all the three patient groups were statistically significant (*p* < 0.0001) (Figure 2(b)). However, between the patient groups, the significance level varied. Between T2DM and T2DM-ATH, the value was *p* > 0.05, whereas between T2DM and ATH groups, *p* values were <0.001. Between ATH and T2DM-ATH groups, the statistical significance was higher (*p* < 0.0001). The mean absorbance values at 410 nm of all the four groups against MG-Fib were as follows: HS (0.187 ± 0.019 (0.161-0.242)), T2DM (0.834 ± 0.088 (0.661-1.040)), ATH (0.759 ± 0.860 (0.617-0.928)), and T2DM-ATH (0.870 ± 0.128 (0.659-1.20)). The coefficient of variation in HS, T2DM, ATH, and T2DM-ATH groups was 10.58%, 10.65%, 11.42%, and 14.73%, respectively (Figure 2(b)). The statistical data are summarized in Table 3.

### 3.5. Affinity of Isolated IgG to N-Fib and MG-Fib

**Figure 3:**
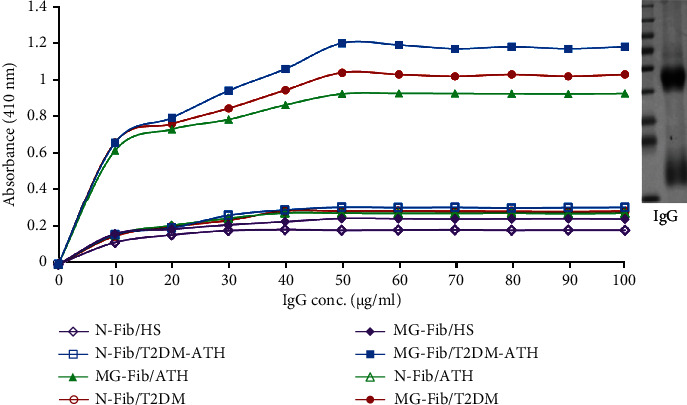
Binding affinity of purified IgG from diabetic (T2DM, *n* = 54), atherosclerosis (ATH, *n* = 33), and diabetic atherosclerosis (T2DM-ATH, *n* = 65) against native fibrinogen (N-Fib) and methylglyoxal- (MG-) glycated fibrinogen (MG-Fib). Insert: the image shows the marker and band (SDS PAGE) of IgG purified from patients' sera.

The IgG concentration increases in T2DM and T2DM-ATH samples with respect to IgG isolated from HS. However, the concentration of IgG from ATH was higher than that from HS but was much lower in comparison to those from T2DM and T2DM-ATH subjects. To evaluate the concentration of IgG needed for the saturation of N-Fib and MG-Fib, direct binding ELISA was performed by isolated IgG from T2DM, ATH, and T2DM-ATH patients (Figure 3). The results revealed that the average saturation concentration of MG-Fib is 50 *μ*g mL^−1^ of IgG from T2DM and T2DM-ATH patients. However, for the ATH group, the saturation concentration was 60 *μ*g mL^−1^.

### 3.6. Competitive ELISA

Competitive/inhibition ELISA was performed to ensure antigen-binding specificity of purified a5utoantibodies that were generated against MG-Fib in patients' sera. The inhibition ELISA was done for 54 IgG samples from the T2DM group, 33 IgG samples from the ATH group, and 65 IgG samples from the T2DM-ATH group, which were screened for greater affinity towards MG-Fib in the direct binding ELISA with sera (Section 3.3). The IgG samples from the HS group were used as a control.

**Figure 4:**
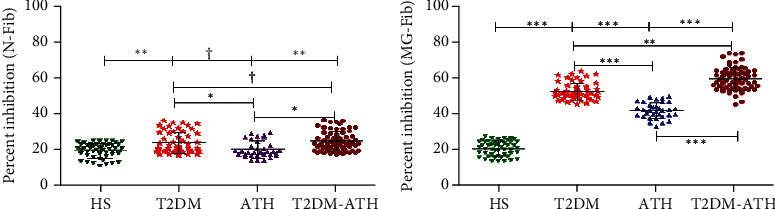
Competitive ELISA. (a) Competitive ELISA of isolated IgG from healthy subjects (HS, *n* = 50) and diabetic (T2DM, *n* = 54), atherosclerosis (ATH, *n* = 33), and diabetic atherosclerosis (T2DM-ATH, *n* = 65) patients with the native immune complex (N-Fib+N-Fib-IgG). T2DM and T2DM-ATH groups show significant percent inhibition with respect to the HS group (^∗∗^*p* < 0.001). The ATH group shows a nonsignificant (^†^*p* > 0.05) percent inhibition with respect to the HS group (^†^*p* > 0.05). Data are presented as mean ± SD of three independent experiments. (b) Competitive ELISA of isolated IgG from healthy subjects (HS, *n* = 50) and diabetic (T2DM, *n* = 54), atherosclerosis (ATH, *n* = 33), and diabetic atherosclerosis (T2DM-ATH, *n* = 65) patients with the glycated immune complex (MG-Fib+MG-Fib-IgG). All the three patient groups showed significant percent inhibition with respect to the HS group and among themselves (^∗^*p* < 0.05, ^∗∗^*p* < 0.001, and ^∗∗∗^*p* < 0.0001). Data are presented as mean ± SD of three independent experiments.

The inhibition ELISA with the native immune complex (N-Fib+IgG) showed statistical significance in the two groups of patients, i.e., T2DM (*p* < 0.001) and T2DM-ATH (*p* < 0.001) with respect to the HS group, whereas HS and ATH groups showed no significance (*p* > 0.05) (Figure 4(a)). At 20 *μ*g mL^−1^ native inhibitor (N-Fib+N-Fib-IgG) concentration, the maximum % inhibition for the T2DM group was found to be 24.50 ± 5.77% (17.76-36.40); for the ATH group, it was 20.88 ± 4.58 (14.52-30.12); and for T2DM-ATH, it was 25.34 ± 5.36 (18.26-37.10). The coefficient of variation in HS, T2DM, ATH, and T2DM-ATH groups was 19.60%, 23.56%, 21.96%, and 21.18%, respectively.

However, with the glycated immune complex (MG-Fib+MG-Fib-IgG), all the three patient groups exhibited high statistical significance (*p* < 0.0001) with respect to the HS group (Figure 4(b)). The maximum % inhibition at 20 *μ*g mL^−1^ inhibitor (MG-Fib+IgG) concentration for the T2DM group was 51.89 ± 4.74 (44.80-62.90); for the ATH group, it was 41.23 ± 4.50 (32.10-49.30); and for the T2DM-ATH group, it was found to be 59.25 ± 6.43 (44.70-73.50). The coefficient of variation in HS, T2DM, ATH, and T2DM-ATH groups was 21.81%, 9.15%, 10.93%, and 10.87%, respectively. The statistical data are summarized in Table 3.

### 3.7. Ketoamine Estimation in Sera of HS and T2DM, ATH, and T2DM-ATH Patients

**Figure 5:**
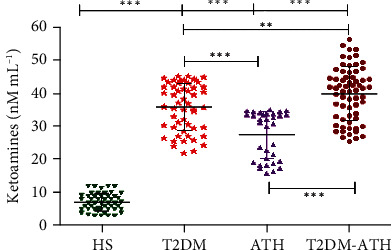
Ketoamine concentration (nM mL^−1^) in the sera of healthy subjects (HS, *n* = 50) and diabetic (T2DM, *n* = 54), atherosclerosis (ATH, *n* = 33), and diabetic atherosclerosis (T2DM-ATH, *n* = 65) patients. The HS group was used as a control. All the three patient groups showed statistical significance with respect to the HS group and among themselves (^∗∗^*p* < 0.001, ^∗∗∗^*p* < 0.0001). Data are presented as mean ± SD of three independent experiments.

In the HS group, the mean ketoamine concentration was found to be 7.15 ± 2.65 nM mL^−1^ (3.12–12.43), whereas in T2DM, ATH, and T2DM-ATH, the mean concentrations of ketoamines were higher up to 36.03 ± 7.30 (21.98–45.32), 27.52 ± 7.16 (16.04–35.19), and 39.83 ± 7.86 nM mL^−1^ (25.51–52.62), respectively. The percent increase in ketoamines in T2DM, ATH, and T2DM-ATH groups with respect to healthy subjects was 80%, 72%, and 82%, respectively (Figure 5). All the three patient groups exhibited high statistical significance (*p* < 0.0001) with respect to HS. Moreover, between the patient groups, the level of significance was from *p* < 0.001 to *p* < 0.0001. The coefficient of variation in HS, T2DM, ATH, and T2DM-ATH groups was 37.06%, 20.28%, 26.04%, and 19.73%, respectively. The statistical data are summarized in Table 4.

### 3.8. AGE Detection in Sera of HS and T2DM, ATH, and T2DM-ATH Patients

**Figure 6:**
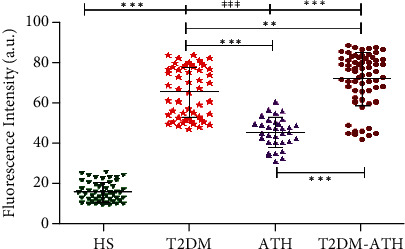
Fluorescence emission profiles at 460 nm upon excitation at 370 nm for the detection of fluorescent AGEs in the sera of healthy subjects (HS, *n* = 50) and diabetic (T2DM, *n* = 54), atherosclerosis (ATH, *n* = 33), and diabetic atherosclerosis (T2DM-ATH, *n* = 65) patients. The HS group was used as a control. All the three patient groups showed statistical significance with respect to the HS group and among themselves (^∗∗^*p* < 0.001, ^∗∗∗^*p* < 0.0001). Data are presented as mean ± SD of three independent experiments.

Fluorescence spectroscopy was performed to detect fluorescent AGEs in HS and patient groups. In the HS group, the mean fluorescence intensity (F.I.) was found to be 16.03 ± 4.78 (a.u.) (9.34–25.47), whereas in T2DM, ATH, and T2DM-ATH, the mean F.I. were higher up to 65.37 ± 12.31 (47.28-83.54), 45.47 ± 7.36 (31.23–60.49), and 71.90 ± 13.13 (a.u.) (42.21–88.61), respectively. The percent increase in F.I. in T2DM, ATH, and T2DM-ATH groups with respect to the HS was 75%, 64%, and 77%, respectively (Figure 6). All the three patient groups exhibited statistical significance (*p* < 0.0001) with the HS group. The coefficient of variation in HS, T2DM, ATH, and T2DM-ATH groups was 29.81%, 18.84%, 16.19%, and 17.91%, respectively. The statistical data are summarized in Table 4.

### 3.9. Protein Carbonyl Estimation in Sera of HS and T2DM, ATH, and T2DM-ATH Patients

**Figure 7:**
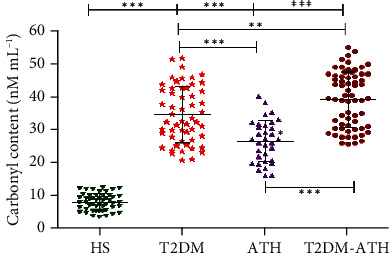
Protein carbonyl concentration (nM mL^−1^) in the sera of healthy subjects (HS, *n* = 50) and diabetic (T2DM, *n* = 54), atherosclerosis (ATH, *n* = 33), and diabetic atherosclerosis (T2DM-ATH, *n* = 65) patients. The HS group was used as a control. All the three patient groups showed statistical significance with respect to the HS group and among themselves (^∗∗^*p* < 0.001, ^∗∗∗^*p* < 0.0001). Data are presented as mean ± SD of three independent experiments.

In the HS group, the mean concentration of protein-bound carbonyls was found to be 7.68 ± 2.61 nM mL^−1^ (3.50-11.60), whereas in T2DM, ATH, and T2DM-ATH, the carbonyl contents were higher up to 34.79 ± 8.59 (20.64–52.05), 26.73 ± 6.40 (16.12–40.16), and 39.52 ± 8.48 nM mL^−1^ (25.75–56.31), respectively. The percent increase in protein-bound carbonyls in T2DM, ATH, and T2DM-ATH groups with respect to HS was 77%, 71%, and 78%, respectively (Figure 7). All the three patient groups exhibit statistical significance (*p* < 0.0001) with respect to the HS group. The coefficient of variation in HS, T2DM, ATH, and T2DM-ATH groups was 34.44%, 24.63%, 23.95%, and 21.25%, respectively. The statistical data are summarized in Table 4.

### 3.10. HMF Estimation in Sera of HS and T2DM, ATH, and T2DM-ATH Patients

**Figure 8:**
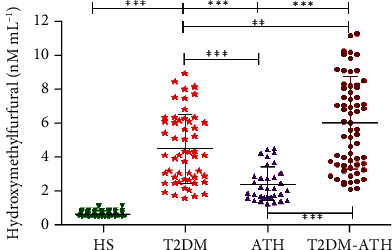
Hydroxymethylfurfural concentration (nM mL^−1^) in the sera of healthy subjects (HS, *n* = 50) and diabetic (T2DM, *n* = 54), atherosclerosis (ATH, *n* = 33), and diabetic atherosclerosis (T2DM-ATH, *n* = 65) patients. The HS group was used as a control. All the three patient groups showed statistical significance with respect to the HS group and among themselves (^∗∗^*p* < 0.001, ^∗∗∗^*p* < 0.0001). Data are presented as mean ± SD of three independent experiments.

In the HS group, the mean concentration of HMF was found to be 0.683 ± 0.17 nM mL^−1^ (0.50–1.14), whereas in T2DM, ATH, and T2DM-ATH, the HMF were higher up to 4.48 ± 2.03 (1.59–8.90), 2.40 ± 1.01 (1.29–4.51), and 6.02 ± 2.67 nM mL^−1^ (2.10–11.21), respectively (Figure 8). The percent increase in HMF in T2DM, ATH, and T2DM-ATH groups with respect to healthy subjects was 84%, 71%, and 88%, respectively. All the three patient groups exhibit statistical significance (*p* < 0.0001) with the HS group. The coefficient of variation in HS, T2DM, ATH, and T2DM-ATH groups was 34.44%, 45.23%, 42.10%, and 44.39%, respectively. The statistical data are summarized in Table 4.

### 3.11. TBARS Estimation in Sera of HS and T2DM, ATH, and T2DM-ATH Patients

**Figure 9:**
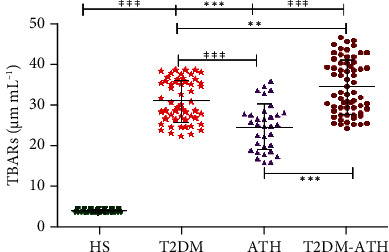
TBARS concentration (*μ*M mL^−1^) in the sera of healthy subjects (HS, *n* = 50) and diabetic (T2DM, *n* = 54), atherosclerosis (ATH, *n* = 33), and diabetic atherosclerosis (T2DM-ATH, *n* = 65) patients. The HS group was used as a control. All the three patient groups showed statistical significance with respect to the HS group and among themselves (^∗∗^*p* < 0.001, ^∗∗∗^*p* < 0.0001). Data are presented as mean ± SD of three independent experiments.

In the HS group, the mean concentration of TBARS was found to be 3.94 ± 0.42 *μ*M mL^−1^ (3.15–4.65), whereas in T2DM, ATH, and T2DM-ATH, the TBARS levels were higher up to 30.94 ± 5.16 (22.17–38.54), 24.62 ± 5.70 (15.87–35.71), and 34.42 ± 6.63 *μ*M mL^−1^ (24.17–46.52), respectively (Figure 9). The percent increase in TBARS in T2DM, ATH, and T2DM-ATH groups with respect to healthy subjects was 87%, 83%, and 88%, respectively. All the three patient groups exhibit statistical significance (*p* < 0.0001) with the HS group. The coefficient of variation in HS, T2DM, ATH, and T2DM-ATH groups was 10.78%, 16.70%, 23.17%, and 19.26%, respectively. The statistical data are summarized in Table 4.

### 3.12. Estimation of Free Lysine in HS and T2DM, ATH, and T2DM-ATH Patients

**Figure 10:**
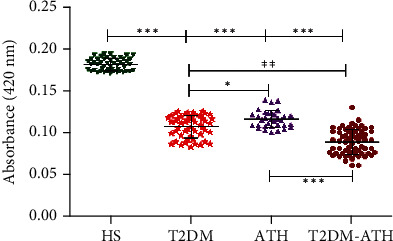
Absorbance profiles of free lysine residues in the sera of healthy subjects (HS, *n* = 50) and diabetic (T2DM, *n* = 54), atherosclerosis (ATH, *n* = 33), and diabetic atherosclerosis (T2DM-ATH, *n* = 65) patients. The HS group was used as a control. All the three patient groups showed statistical significance with respect to the HS group and among themselves (^∗^*p* < 0.05, ^∗∗^*p* < 0.001, and ^∗∗∗^*p* < 0.0001). Data are presented as mean ± SD of three independent experiments.

In the HS group, the mean absorbance at 420 nm was found to be 0.181 ± 0.007 (0.017–0.194), whereas in T2DM, ATH, and T2DM-ATH, the absorbance was lower up to 0.107 ± 0.012 (0.083–0.125), 0.116 ± 0.010 (0.100–0.139), and 0.088 ± 0.014 (0.061–0.130), respectively. The percent decrease in free lysine in T2DM, ATH, and T2DM-ATH groups with respect to healthy subjects was 40%, 35%, and 51%, respectively (Figure 10). All the three patient groups exhibit statistical significance (*p* < 0.0001) with the HS group. The coefficient of variation in HS, T2DM, ATH, and T2DM-ATH groups was 4.07%, 12.08%, 8.77%, and 16.85%, respectively. The statistical data are summarized in Table 4.

### 3.13. Estimation of Free Arginine in HS and T2DM, ATH, and T2DM-ATH Patients

**Figure 11:**
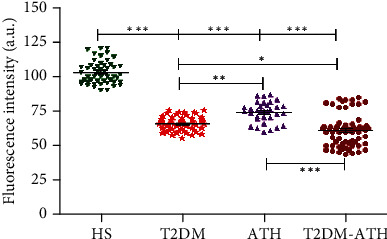
Fluorescence emission profiles at 395 nm upon excitation at 280 nm for the detection of free arginine residues in the sera of healthy subjects (HS, *n* = 50) and diabetic (T2DM, *n* = 54), atherosclerosis (ATH, *n* = 33), and diabetic atherosclerosis (T2DM-ATH, *n* = 65) patients. The HS group was used as a control. All the three patient groups showed statistical significance with respect to the HS group and among themselves (^∗^*p* < 0.05, ^∗∗^*p* < 0.001, and ^∗∗∗^*p* < 0.0001). Data are presented as mean ± SD of three independent experiments.

In the HS group, the mean emission fluorescence intensity (F.I.) at 395 nm was found to be 102.91 ± 8.18 (a.u.) (90.17–120.33), whereas in T2DM, ATH, and T2DM-ATH, the mean F.I. were lower up to 66.21 ± 5.11 (55.28–75.63), 74.44 ± 7.39 (60.12–86.75), and 61.39 ± 11.54 (a.u.) (44.09–84.91), respectively (Figure 11). The percent decrease in free arginine in T2DM, ATH, and T2DM-ATH groups with respect to healthy subjects was 35%, 27%, and 40%, respectively. All the three patient groups exhibit statistical significance (*p* < 0.0001) with respect to the HS group. The coefficient of variation in HS, T2DM, ATH, and T2DM-ATH groups was 7.95%, 7.72%, 9.94%, and 18.71%, respectively. The statistical data are summarized in Table 4.

### 3.14. CML Estimation in Sera of HS and T2DM, ATH, and T2DM-ATH Patients

**Figure 12:**
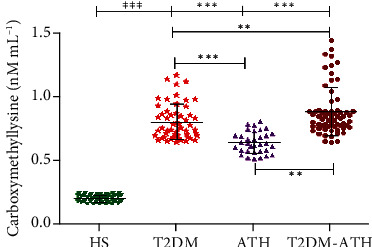
Carboxymethyllysine concentration (nM mL^−1^) in the sera of healthy subjects (HS, *n* = 50) and diabetic (T2DM, *n* = 54), atherosclerosis (ATH, *n* = 33), and diabetic atherosclerosis (T2DM-ATH, *n* = 65) patients. The HS group was used as a control. All the three patient groups showed statistical significance with respect to the HS group and among themselves (^∗^*p* < 0.05, ^∗∗^*p* < 0.001, and ^∗∗∗^*p* < 0.0001). Data are presented as mean ± SD of three independent experiments.

In the HS group, the mean concentration of CML was found to be 0.215 ± 0.026 nM mL^−1^ (0.170–0.257), whereas in T2DM, ATH, and T2DM-ATH, the CML were higher up to 0.814 ± 0.141 (0.654–1.159), 0.653 ± 0.084 (0.524–0.821), and 0.889 ± 0.186 nM mL^−1^ (0.653–1.447), respectively (Figure 12). The percent increase in CML concentration in T2DM, ATH, and T2DM-ATH groups with respect to healthy subjects was 73%, 67%, and 75%, respectively. All the three patient groups exhibit statistical significance (*p* < 0.0001) with respect to the HS group. The coefficient of variation in HS, T2DM, ATH, and T2DM-ATH groups was 12.31%, 17.36%, 12.95%, and 21.34%, respectively. The statistical data are summarized in Table 4.

## 4. Discussion

Reducing sugars along with dicarbonyls or reactive carbonyl species (RCS) such as methylglyoxal (MG) can instigate *in vivo* nonenzymatic glycosylation [18]. This reaction is a slow process but increases several folds during prolonged and persistent hyperglycemia [4]. It has been investigated that MG is a highly reactive dicarbonyl that leads to cellular dysfunction by perturbing native structure and normal function of proteins. Moreover, in the persistent hyperglycemic state, the concentration of dicarbonyls was significantly elevated [11]. Furthermore, exposure and generation of neoepitopes on glycated proteins render them highly immunogenic which leads to the production of autoantibodies [44].

The direct binding ELISA of patients' sera has revealed a higher prevalence of autoantibodies in type 2 diabetes mellitus (T2DM, 54%), atherosclerosis (ATH, 33%), and diabetic atherosclerosis (T2DM-ATH, 65%) against MG-Fib. However, healthy subject (HS) sera did not show significant binding with both the N-Fib and MG-Fib. From our *in vitro* characterization study, it was implied that MG-mediated glycation of fibrinogen leads to the formation of fibrinogen-AGEs (Fib-AGEs), which might elicit immune response via neoepitope generation [38]. The presence of highly specific anti-MG-Fib autoantibodies or anti-Fib-AGEs autoantibodies in the sera of T2DM (54%), ATH (33%), and T2DM-ATH (65%) patients is in agreement with our *in vitro* study. Moreover, direct binding ELISA of sera against N-Fib also showed significant binding in some samples of T2DM and T2DM-ATH patient groups. The reason might be the denaturation and exposure of neoepitopes on N-Fib during incubation at 37°C for 7 days.

The direct binding of isolated IgG from those patients' sera that showed higher affinity towards MG-Fib further validates the generation of autoantibodies (anti-MG-Fib-IgG) in the patient groups. Moreover, autoantibodies (anti-N-Fib-IgG) against N-Fib were also found in all the three patient groups and showed significant binding with respect to the HS group. However, the absorbance at 410 nm was much lower in N-Fib with respect to MG-Fib. The results are in agreement with direct binding ELISA of sera against N-Fib.

In competitive/inhibition ELISA, the maximum inhibition of isolated IgG against N-Fib and MG-Fib follows the same descending order: T2DM − ATH > T2DM > ATH. However, in the case of N-Fib, the maximum percent inhibition among T2DM and T2DM-ATH patient groups with respect to the HS group was found to be significant (*p* < 0.05), whereas maximum percent inhibition against MG-Fib in the patient groups with respect to the HS group was significant (*p* < 0.05). Thus, the results suggest that the high specificity of serum autoantibodies or isolated IgG towards MG-Fib is due to the generation of neoepitopes on MG-modified fibrinogen as compared to N-Fib. Moreover, percent inhibition towards N-Fib reveals that N-Fib gets denatured during incubation and neoepitopes are exposed, against which autoantibodies (anti-N-Fib-IgG) are generated.

The NBT reduction assay of patients' sera suggests a 5-6-fold increase in ketoamine concentration in T2DM and T2DM-ATH patients with respect to healthy subjects. However, in ATH patients, ~3-fold increase in ketoamines was noticed in comparison to that in healthy individuals. The early glycation products reduce the yellow NBT to purple monoformazan. It has been suggested that NBT is reduced by the action of superoxide radicals that are formed during the degradation of ketoamines [61, 62]. Furthermore, the superoxide radicals via the Fenton reaction give rise to hydroxyl radicals. Thus, it is hypothesized that both superoxide and hydroxyl radicals might cause damage to protein molecules besides glycation [63, 64].

The fluorescence emission profiles at 460 nm confirmed the presence of AGEs in T2DM, ATH, and T2DM-ATH patients' sera. In comparison to healthy individuals, the patient groups exhibit a 2-4-fold increase in fluorescence intensities (F.I.). However, in the ATH group, there is a ~10% decline in F.I. with respect to T2DM and T2DM-ATH groups which indicates more generation of fluorescent AGEs under a hyperglycemic state. Moreover, it has been reported that the fluorescence is exhibited only by those AGEs that have heterocyclic structures [65, 66].

Protein carbonyls are the markers of oxidative stress formed via enediol reaction from ketoamines (67, 68). During enediol reaction, superoxide radicals are formed that give rise to hydroxyl radicals via the Fenton reaction that further contributes to oxidative stress (69, 70). In our study, 3-5-fold increase in carbonyl contents in T2DM, ATH, and T2DM-ATH patients confirms higher oxidative stress. However, in the ATH group, the carbonyl contents were lower in concentration than those in T2DM and T2DM-ATH further authenticating hyperglycemia-induced oxidative stress in diabetic subjects. Earlier studies on diabetic patients suggest that increased accumulation of carbonyl contents exerts an adverse effect on physiological systems (71, 72). Both hydroxymethylfurfural (HMF) and malondialdehyde (MDA) are thiobarbituric acid (TBA) reactive species (TBARS) (38). HMF is released upon hydrolysis of ketoamines by weak acids, whereas MDA is an indicator of oxidative stress generated during oxidation of proteins to protein carbonyls via enediol reaction (60, 73). In our study, we found a 3-8-fold increase in HMF content and a 6-8-fold increase in MDA concentration in T2DM, ATH, and T2DM-ATH patients with respect to healthy subjects. However, in the ATH group, the HMF contents and MDA concentration were lesser than those in T2DM and T2DM-ATH. This indicates that more oxidative stress is generated during diabetes-induced prolonged and persistent hyperglycemia. The results indicate enhanced oxidative stress in diabetic and diabetes atherosclerosis patients due to hyperglycemia-mediated accelerated protein glycation. The ketoamines, carbonyl contents, HMF, and TBARS results are in agreement with each other.

The lysine (Lys) and arginine (Arg) amino acids are more susceptible to glycation because of the presence of free amino group (-NH_2_) and guanidino group in their respective side chains (59). In our study, the significant drop in free Lys and free Arg in T2DM and T2DM-ATH patients with respect to the HS group portrays the extent of glycation and AGE formation. The percentage of reacted lysine and arginine residues in T2DM and T2DM-ATH patients is much higher with respect to healthy individuals.

CML is a nonfluorogenic AGE formed in hyperglycemic individuals (74–76). In our study, a 3-4-fold increase in CML levels in the patient groups suggests increased glycative stress in T2DM and T2DM-ATH patients in comparison to healthy individuals.

## 5. Conclusion

The present study explored methylglyoxal-mediated structural perturbations induced in fibrinogen protein that results in the generation of neoepitopes, which in turn provokes an immune response in the form of autoantibodies (anti-MG-Fib antibodies) in T2DM, ATH, and T2DM-ATH patients. However, the precise role of glycated fibrinogen in the pathogenesis of diabetes-associated micro- and macrovascular complications is still in veil and needs further investigation. We hope that our findings may provide an approach to predict diabetes-related atherosclerosis before its onset by validating glycated fibrinogen as a new biomarker. Moreover, this study might facilitate the scientific fraternity to minimize the menace of glycation-induced oxidative and carbonyl stress, by inhibiting and scavenging the intermediates of nonenzymatic glycosylation.

## Figures and Tables

**Table 1:** Comparative physicochemical characterization of fibrinogen protein upon glycation with 5 mM and 10 mM of methylglyoxal (MG).

Characterization	Fibrinogen		
	Native	5 mM MG	10 mM MG
UV-Vis (280 nm)	0.231 ± 0.05	1.134 ± 0.24 79% ↑	1.268 ± 0.42 81% ↑
Ketoamines (nM mL^−1^)	4.43 ± 0.76	38.68 ± 4.3 88% ↑	46.28 ± 6.9 90% ↑
AGEs (*λ*_ex_—370; *λ*_em_—460)	3.17 ± 0.5	12.67 ± 3.3 74% ↑	15.73 ± 4.7 79% ↑
Intrinsic fluorescence (*λ*_ex_—280; *λ*_em_—340)	304 ± 41	77.31 ± 10 74% ↓	55.30 ± 6.2 81% ↓
Circular dichroism (mdeg)	−10.23 ± 0.1	−15.4 ± 1.3	−17.3 ± 1.7
FTIR (cm^−1^)	1636 3438	1640 3442	1638 3444
Carbonyl content (nM mg^−1^)	40 ± 10	147 ± 18 72% ↑	219 ± 31 81% ↑
HMF (nmol mL^−1^)	1.2 ± 0.2	5.8 ± 0.78 79% ↑	7.2 ± 1.6 83% ↑
TBARS (*μ*M mL^−1^)	2.2 ± 0.3	9 ± 0.98 75% ↑	11.6 ± 1.2 81% ↑
Free lysine (420 nm)	0.423 ± 0.02	0.098 ± 0.033 76% ↓	0.079 ± 0.02 81% ↓
Free arginine (*λ*_ex_—312; *λ*_em_—395)	149 ± 18	69.69 ± 13.4 53% ↓	57.73 ± 9.1 61% ↓
CML (nM mg^−1^)	183 ± 28	631 ± 76 70% ↑	657 ± 54 72% ↑
ThT (*λ*_ex_—440; *λ*_em_—485)	32.31 ± 9.1	107 ± 11 69% ↑	131 ± 20 75% ↑

All the data is presented as mean ± standard deviation (SD). Statistical significance of data was determined by Student's *t*-test, and a *p* value of <0.05 was considered significant. The upward arrows (**↑**) represent percent increase whereas downward arrows (**↓**) represent percent decrease.

**Table 2:** Clinical and metabolic parameters of the T2DM, ATH, and T2DM-ATH patients.

Characteristics	HS	T2DM	ATH	T2DM-ATH
Sample size	50	100	100	100
Age (years)	30 ± 8	53.4 ± 11.4	59 ± 7.1	55.4 ± 10.24
Gender (M/F)	25/25	67/33	82/18	78/12
DD (years)	—	7.6 ± 2.3	8.1 ± 4.2	9.2 ± 5.2
BMI (kg m^−2^)	20.5 ± 1.5	26.55 ± 2.13^∗∗^	21.6 ± 2^∗^	27.3 ± 2.3^∗∗^
BP (SST) (mmHg)	120 ± 15	125 ± 12^†^	164 ± 22.0^∗∗^	180 ± 9.5^∗∗^
BP (DST) (mmHg)	65 ± 7	78 ± 9^†^	106 ± 10^∗∗^	110 ± 12^∗∗^
Glucose: fasting (mg dL^−1^)	88 ± 8	196 ± 8.2^∗∗^	90 ± 6	222 ± 16^∗∗∗^
HbA1c (%)	4.5 ± 0.14	6.6 ± 1.9^∗∗^	4.4 ± 0.2	7.1 ± 2.9^∗∗∗^
TC (mg dL^−1^)	160 ± 2.1	175 ± 2.3^†^	308 ± 3.2^∗∗∗^	284 ± 10.4^∗∗^
LDL (mg dL^−1^)	144 ± 4.11	147 ± 0.1^†^	244 ± 2.5^∗∗∗^	246 ± 4.4^∗∗^
HDL (mg dL^−1^)	48 ± 1.8	37.8 ± 3.9^†^	33 ± 1.3^∗∗^	27 ± 4.6^∗∗^
TG (mg dL^−1^)	176 ± 4.1	184 ± 6.8^∗^	300 ± 3.3^∗∗∗^	204 ± 5.8^∗∗^

Values are expressed as mean ± SD: significance/nonsignificance level: ^†^*p* > 0.05 (not significant), ^∗^*p* < 0.05 (significant), ^∗∗^*p* < 0.001 (highly significant), and ^∗∗∗^*p* < 0.0001 (extremely significant). All the parameters were tested by one-way ANOVA followed by the post hoc test and Dunnett *t*-test using the Statistical Package for the Social Sciences (SPSS) package, version 20.0. HS: healthy subjects; M/F: male/female; BMI: body mass index; DD: duration of disease; SST: systolic; DST: diastolic; HbA1c: glycosylated hemoglobin; TC: total cholesterol; LDL: low-density lipoprotein; HDL: high-density lipoprotein; TG: triglycerides.

**Table 3:** Statistical data showing direct binding and inhibition ELISA of sera and isolated IgG from HS, T2DM, ATH, and T2DM-ATH patients' groups against native fibrinogen (N-Fib) and methylglyoxal-glycated fibrinogen (MG-Fib).

Disease	*N*	Mean	SD	Min.	Max.	CV	*p* value
Direct binding ELISA (N-Fib/sera)							
HS	50	0.204	0.021	0.175	0.234	10.63%	…..
T2DM	100	0.215	0.024	0.192	0.288	11.50%	*p* < 0.05
ATH	100	0.212	0.029	0.180	0.282	13.78%	*p* > 0.05
T2DM-ATH	100	0.220	0.021	0.200	0.295	09.71%	*p* < 0.001
Direct binding ELISA (MG-fib/sera)							
HS	50	0.211	0.018	0.182	0.240	8.81%	…..
T2DM	100	0.513	0.209	0.264	0.908	40.70%	*p* < 0.0001
ATH	100	0.424	0.170	0.280	0.846	40.09%	*p* < 0.0001
T2DM-ATH	100	0.581	0.223	0.266	0.970	38.37%	*p* < 0.0001
Direct binding ELISA (N-Fib/IgG)							
HS	50	0.186	0.022	0.163	0.235	12.16%	—
T2DM	54	0.231	0.046	0.178	0.338	20.19%	*p* < 0.05
ATH	33	0.225	0.050	0.167	0.330	22.31%	*p* < 0.05
T2DM-ATH	65	0.238	0.050	0.180	0.370	21.85%	*p* < 0.001
Direct binding ELISA (MG-Fib/IgG)							
HS	50	0.187	0.019	0.161	0.242	10.58%	—
T2DM	54	0.834	0.088	0.661	1.040	10.65%	*p* < 0.0001
ATH	33	0.759	0.086	0.617	0.928	11.42%	*p* < 0.0001
T2DM-ATH	65	0.870	0.128	0.659	1.200	14.73%	*p* < 0.0001
Inhibition ELISA (N-Fib)							
HS	50	20.28	3.97	11.88	25.80	19.60%	—
T2DM	54	24.50	5.77	17.76	36.40	23.56%	*p* < 0.001
ATH	33	20.88	4.58	14.52	30.12	21.96%	*p* > 0.05
T2DM-ATH	65	25.34	5.36	18.26	37.10	21.18%	*p* < 0.001
Inhibition ELISA (MG-Fib)							
HS	50	19.78	4.18	12.40	26.70	21.18%	—
T2DM	54	51.89	4.74	44.80	62.90	9.15%	*p* < 0.0001
ATH	33	41.23	4.50	32.10	49.30	10.93%	*p* < 0.0001
T2DM-ATH	65	59.25	6.43	44.70	73.50	10.87%	*p* < 0.0001

The data were presented as mean ± SD, and the statistical significance level (*p* < 0.05) between HS, T2DM, ATH, and T2DM-ATH groups was determined by one-way ANOVA. All the three patient groups exhibit statistical significance (*p* < 0.0001) with the HS group. “*N*” is the no. of subjects in each group, “Min.” is the minimum value, “Max.” is the maximum value, “SD” is the standard deviation, “CV” is the coefficient of variation, and “*p*” is the level of significance. The upward arrows (↑) represent percent increase whereas downward arrows (**↓**) represent percent decrease in parameters with respect to the HS group.

**Table 4:** Statistical data showing ketoamines, AGEs, protein carbonyls, hydroxymethyl furfural (HMF), TBARS, free lysine, free arginine, and carboxymethyllysine concentrations in the sera of HS, T2DM, ATH, and T2DM-ATH patient groups.

Disease	*N*	Mean	SD	Min.	Max.	CV	*p* value	% Inc/Dec
Ketoamines								
HS	50	7.15	2.65	3.12	12.43	37.06%	—	
T2DM	54	36.03	7.30	21.98	45.32	20.28%	*p* < 0.0001	80% ↑
ATH	33	27.52	7.16	16.04	35.19	26.04%	*p* < 0.0001	72% ↑
T2DM-ATH	65	39.83	7.86	25.51	52.62	19.73%	*p* < 0.0001	82% ↑
AGEs								
HS	50	16.03	4.78	9.34	25.47	29.81%	—	
T2DM	54	65.37	12.31	47.28	83.54	18.84%	*p* < 0.0001	75% ↑
ATH	33	45.47	7.36	31.23	60.49	16.19%	*p* < 0.0001	64% ↑
T2DM-ATH	65	71.90	13.13	42.21	88.61	17.91%	*p* < 0.0001	77% ↑
Carbonyl content								
HS	50	7.68	2.61	3.50	11.60	34.44%	—	
T2DM	54	34.79	8.56	20.64	52.05	24.63%	*p* < 0.0001	77% ↑
ATH	33	26.73	6.40	16.12	40.16	23.95%	*p* < 0.0001	71% ↑
T2DM-ATH	65	39.52	8.48	25.75	56.31	21.25%	*p* < 0.0001	78% ↑
Hydroxymethyl furfural								
HS	50	0.68	0.17	0.50	1.14	25.08%	—	
T2DM	54	4.48	2.03	1.59	8.90	45.23%	*p* < 0.0001	84% ↑
ATH	33	2.40	1.01	1.29	4.51	42.10%	*p* < 0.0001	71% ↑
T2DM-ATH	65	6.02	2.67	2.10	11.21	44.39%	*p* < 0.0001	88% ↑
TBARS								
HS	50	3.94	0.42	3.15	4.65	10.78%	—	
T2DM	54	30.94	5.16	22.17	38.54	16.70%	*p* < 0.0001	87% ↑
ATH	33	24.62	5.70	15.87	35.71	23.17%	*p* < 0.0001	83% ↑
T2DM-ATH	65	34.42	6.63	24.17	46.52	19.26%	*p* < 0.0001	88% ↑
Free lysine								
HS	50	0.181	0.007	0.171	0.194	4.07%	—	
T2DM	54	0.107	0.012	0.083	0.125	12.08%	*p* < 0.0001	40% ↓
ATH	33	0.116	0.010	0.100	0.139	8.77%	*p* < 0.0001	35% ↓
T2DM-ATH	65	0.088	0.014	0.061	0.130	16.85%	*p* < 0.0001	51% ↓
Free arginine								
HS	50	102.91	8.18	90.17	120.33	7.95%	—	
T2DM	54	66.21	5.11	55.28	75.63	7.72%	*p* < 0.0001	35% ↓
ATH	33	74.44	7.39	60.12	86.75	9.94%	*p* < 0.0001	27% ↓
T2DM-ATH	65	61.39	11.54	44.09	84.91	18.17%	*p* < 0.0001	40% ↓
Carboxymethyllysine								
HS	50	0.215	0.026	0.170	0.257	12.31%	—	
T2DM	54	0.814	0.141	0.654	1.159	17.36%	*p* < 0.0001	73% ↑
ATH	33	0.653	0.084	0.524	0.821	12.95%	*p* < 0.0001	67% ↑
T2DM-ATH	65	0.889	0.186	0.653	1.447	21.34%	*p* < 0.0001	75% ↑

The data was presented as mean ± SD, and the statistical significance level (*p* < 0.05) between HS, T2DM, ATH, and T2DM-ATH groups was determined by one-way ANOVA. All the three patient groups exhibit statistical significance (*p* < 0.0001) with the HS group. “*N*” is the no. of subjects in each group, “Min.” is the minimum value, “Max.” is the maximum value, “SD” is the standard deviation, “CV” is the coefficient of variation, and “*p*” is the level of significance. The upward arrows (↑) represent percent increase whereas downward arrows (**↓**) represent percent decrease in parameters with respect to the HS group.

## Data Availability

Data is available within the text of the manuscript.

## Abbreviations

MG: Methylglyoxal
Fib: Fibrinogen
N-Fib: Native fibrinogen
MG-Fib: MG-glycated fibrinogen/or MG-modified fibrinogen
N-Fib-IgG: Anti-N-Fib autoantibodies
MG-Fib-IgG: Anti-MG-fib autoantibodies
N-Fib+N-Fib-IgG: Native immune complex
MG-Fib+MG-Fib-IgG: Glycated immune complex
HS: Healthy subjects
T2DM: Type 2 diabetes mellitus
ATH: Atherosclerosis
T2DM-ATH: Diabetic atherosclerosis.
